# Exploring the Catalytic Efficiency of Lithium Bis(trimethylsilyl)amide (LiHMDS) in Lactide Polymerization

**DOI:** 10.3390/polym17030429

**Published:** 2025-02-06

**Authors:** Almas Kiran, Achukee Chinedu Kingsley, Hassan Ahmed

**Affiliations:** 1 University of Chinese Academy of Sciences, Beijing 100049, China; rahiba.bhatti@yahoo.com (A.K.); nedugunner252@gmail.com (A.C.K.); 2 Key Laboratory of Photoelectric Conversion and Utilization of Solar Energy, Qingdao Institute of Bioenergy and Bioprocess Technology, Chinese Academy of Sciences, Qingdao 266101, China; 3 Shandong Energy Institute, Qingdao 266101, China

**Keywords:** biodegradable, lithium-based metal catalyst, cyclic esters, ring-opening polymerization, poly(lactic acid)

## Abstract

The exploration of efficient catalysts for the ring-opening polymerization of cyclic esters has significant implications for the synthesis of biocompatible and biodegradable polymers. In this work, the simple catalyst lithium bis(trimethylsilyl)amide (LiHMDS) with high activity was explored in detail for the synthesis of polylactide (PLA). Using LiHMDS as the catalyst, various cyclic esters were polymerized to obtain diverse sustainable polyesters, such as poly(lactide), poly(δ-valerolactone), and poly(caprolactone), with controlled molecular weights and narrow molecular weight distributions. PLA synthesis was accomplished in just a few minutes at room temperature, contributing to the sustainable advancement of this polymer.

## 1. Introduction

Petrochemical-based synthetic polymers are integral to nearly every facet of modern life, from packaging materials to automotive components and medical devices. However, the widespread use of these polymers has resulted in significant environmental issues due to their non-biodegradability and prolonged persistence in ecosystems. The accumulation of plastic waste in landfills and oceans has led to increased awareness and concern about the environmental impacts of these materials [1,2,3,4,5,6]. As a result, there is a growing demand for sustainable alternatives that can reduce the reliance on nonrenewable resources and mitigate environmental degradation. Among the promising alternatives, bio-based polyesters have gained considerable attention for their potential to replace traditional synthetic polymers. Polylactide (PLA), in particular, has emerged as a standout material in this area due to its exceptional mechanical and physical properties. PLA is derived from renewable resources such as corn starch or sugarcane, making it an eco-friendly alternative to petrochemical-based plastics. Its versatility and performance characteristics, including good tensile strength, transparency, and biodegradability, make it especially valuable for a wide range of applications, particularly in the pharmaceutical and medical fields, where its biocompatibility and safety are highly beneficial [7,8].

It is well known that the physical and mechanical properties of PLA are directly influenced by its stereochemistry. Therefore, ring-opening polymerization (ROP) is a highly efficient method for producing PLA with well-defined structures, often using metal-based initiators [9,10,11]. For instance, Lithium bis(trimethylsilyl)amide (LiHMDS) has exceptional flexibility and nucleophilicity, which makes it very suitable for initiating the ring-opening polymerization of various polyesters [12,13,14,15,16]. This catalyst demonstrates an exciting feature in which the exchange reaction between the active substance and LiHMDS molecules can lead to the formation of a zigzag chain polymer [17,18,19,20]. Liu’s group reported that using Lithium bis(trimethylsilyl)amide can facilitate rapid polymerization, generating specific polypeptides with low dispersity over a wide range of average chain lengths, thereby enabling parallel and large-scale synthesis [21,22,23,24]. In the realm of ring-opening polymerization (ROP) of cyclic esters, various catalysts play a pivotal role in determining the efficiency and control of the polymerization process. For example, the organic bases 1,5,7-triazabicyclo [4.4.0]dec–5-ene (TBD), 7-methyl-1,5,7-triazabicyclo[4.4.0]dec–5-ene (MTBD), 1,8-diazabicyclo[5.4.0]undec–7-ene (DBU), and 4-(N,N-dimethylamino)pyridine (DMAP) have been found to be highly effective in polymerizing cyclic esters such as lactide, γ-valerolactone, and ε-caprolactone, as demonstrated by Hedrick, Waymouth, and their colleagues [25,26]. While TBD provides high catalytic activity, it often results in reduced control over polymerization kinetics for highly reactive monomers like lactide (LA). On the other hand, DBU combined with thiourea systems, although offering finer control, require prolonged reaction times and may lead to incomplete monomer conversion [27]. Therefore, it is of great significance to develop applicable M(HMDS) catalysts for the ROP of cyclic esters.

In recent work, our group explored the use of readily accessible M(HMDS) (M = Li, Na, and K) catalysts to facilitate the alcoholysis of polylactic acid (PLA) [28,29]. These metal-based catalysts have shown promising results in efficiently recycling mixed PLA waste materials, making them an attractive option for sustainable processes. However, one of the major challenges in using M(HMDS) catalysts for ring-opening polymerization lies in the difficulty of precisely controlling the molecular weight distribution and polymer microstructure. This limitation arises due to the fast initiation and propagation rates inherent in these systems. In this study, we focused on commercially available lithium bis(trimethylsilyl)amide (LiHMDS) as the catalyst for the ROP of lactide and other lactones. The results demonstrated that LiHMDS exhibits remarkable activity in the polymerization of lactide, enabling the synthesis of polymers with well-defined molecular weights and narrow polydispersity at mild reaction conditions.

## 2. Materials and Methods

### 2.1. Materials

L-Lactide (99%) was purchased from Heowns (Tianjin, China) and underwent three recrystallization cycles from anhydrous toluene, forming white crystals. δ-Valerolactone (>98%, TCI) and ε-Caprolactone (>99.9%, TCI) were distilled over CaH_2_ under an argon atmosphere. Lithium bis(trimethylsilyl)amide (LiHMDS) (1 M in THF) was purchased from Energy Chemical (Nottinghamshire, UK), and benzyl alcohol (BnOH, >99.9%), from Sigma-Aldrich (Saint Louis, MO, USA). The BnOH dissolved into a 1 M solution of toluene as the initiator. Benzhydrol (99%) was purchased from Macklin Biochemical Co., Ltd. (Shanghai, China) and dried in vacuum overnight. Benzoic acid (>99.9%, Sigma-Aldrich) in CDCl_3_ was used as the quenching solution. All dry solvents were redistilled after being collected in the solvent purification system and then stored over molecular sieves (4A) in a glove box for no longer than one month.

### 2.2. Characterizations

Nuclear magnetic resonance measurements (^1^H NMR, ^13^C NMR) were conducted at room temperature using a Bruker Advance apparatus (Bruker, Billerica, MA, USA) operating at 400 MHz. CDCl_3_ is employed as the internal reference. The molecular weights (M_n_) and dispersity (Ð) of the polymers were measured using gel permeation chromatography (GPC) with an Agilent 1260 LC system from the United States (Agilent Technologies, Santa Clara, CA, USA). The eluent was tetrahydrofuran (THF) with a flow rate of 1–2 mL/min at 40 °C, maintaining the sample concentration at 1 mg/mL. Differential scanning calorimetry (DSC) measurements were performed on the DSC 3500 Sirius. The temperature was calibrated with C_10_H_16_, indium, tin, bismuth, and the zinc standard. Measurements were performed under N_2_ atmosphere with a flow rate of 20 mL/min. Each sample, with a mass of 10 mg, was used for the measurement. Following standard procedures, the samples were first heated from 20 °C to 250 °C at a heating rate of 10 K/min. In the second heating scan, samples were cooled to 0 °C at 10 K/min and kept at 0 °C for 20 min to eliminate any thermal history, and subsequently reheated to 250 °C at 10 K/min.

### 2.3. Typical Ring-Opening Polymerization of L-Lactide (L-LA)

A 5 mL glass of Schlenk was dried and put into a glove box under an argon atmosphere. To initiate the polymerization process, Lithium bis(trimethylsilyl)amide (1.673 mg, 10 µmol, 1 equiv.) was dissolved in toluene (1.0 mL) and added to a Schlenk tube along with the initiator, BnOH solution (20 μL, 20 µmol, 2 equiv.). The mixture was stirred for some minutes, after which L-LA (144 mg, 1 mmol, 100 equiv.) was introduced. The polymerization process was performed at room temperature (25 ± 5 °C) and quenched by adding benzoic acid solution (1 M in CDCl_3_) at a specified time. The monomer conversion was calculated by comparing the integration of the methyl signals from the unreacted monomer to the methyl area of the polymer using the ^1^H NMR spectrum. The solvent was removed by vacuum evaporation, and the polymer was purified and isolated by precipitating it in cold methanol (MeOH), followed by rapid drying under reduced pressure.

A similar polymerization protocol was followed for the ring-opening polymerization of δ-valerolactone and ε-caprolactone using Ph_2_CHOH as the initiator.

## 3. Results and Discussion

First, we are highly motivated to identify a biocompatible and non-toxic catalyst that offers performance equal to or better than that of stannous octoate, which is commonly used for the synthesis of PLA. Table 1 shows the results of the ring-opening polymerization of the enantiopure L-Lactide using LiHMDS with various initiators at different monomer-to-catalyst loadings.

Initiating the polymerization reaction using lithium LiHMDS alone as a catalyst demonstrated low activity in the ring-opening polymerization (ROP) of L-lactide. Under these conditions, the reaction was sluggish, requiring 12 h to achieve a conversion of 98% (Table 1, entry 1). The resulting polymer exhibited a relatively broad polydispersity (*Ð* = 1.55). These observations imply that the amide groups in LiHMDS alone are insufficient to serve as effective initiators, likely because they do not facilitate an efficient nucleophilic attack on the lactide monomer. These challenges emphasize the need for an external initiator that can enhance the reaction kinetics and allow for better control over the polymerization process. To address this issue, we introduced benzyl alcohol (BnOH) as the initiator, which significantly improved the catalytic activity. The reaction time was dramatically reduced from 12 h to just 30 min, and the polymerization achieved a conversion of 92% (Table 1, entry 2). This dramatic improvement in polymerization efficiency is attributed to the ability of the alcohol group in BnOH to activate the lactide monomer. The hydroxyl group in the alcohol facilitates a more efficient nucleophilic attack on the lactide, leading to a faster initiation and propagation step in the polymerization process (see Figure S27). The result is not only a faster reaction but also a polymer with improved control over the molecular weight and a narrower distribution.

Further optimization was performed by increasing the ratio of BnOH to two equivalents, which resulted in an even more efficient polymerization. The reaction time was reduced to just 8 min, with a conversion rate of 93% (Table 1, entry 3). This improvement can be attributed to the increased availability of the alcohol initiator, which promotes a more controlled polymerization process. With a higher initiator concentration, the polymerization proceeds more rapidly and the polymer chains grow at a more uniform rate, leading to a narrower molecular weight distribution. This trend continued when the ratio of BnOH was increased further to four equivalents. At this concentration, the reaction was completed in only 3 min, with a conversion rate of 90% (Table 1, entry 4). Notably, the polydispersity also decreased with increasing BnOH concentration. At the highest initiator loading (four equivalents), the polydispersity reached *Ð* = 1.10, suggesting a high level of control over the polymerization process and a well-defined final product. These results strongly demonstrate the significant role of BnOH in enhancing the catalytic activity of LiHMDS and improving the precision of the polymerization.

Interestingly, when the initiator was switched to diphenylmethanol (Ph_2_CHOH), similar results were obtained, indicating that the nature of the alcohol initiator does not significantly affect the polymerization activity or control under the same conditions. This observation suggests that LiHMDS is effective with a variety of alcohol-based initiators, which broadens the potential application of this catalyst in different polymerization scenarios. As shown in Table 1 (entries 5–7), increasing the ratio of Ph_2_CHOH also led to improved reaction efficiency and better control over the polymerization. For instance, when four equivalents of Ph_2_CHOH were used as the initiator, the resulting polymer exhibited a narrow polydispersity (Table 1, entry 7). This indicates that increasing the concentration of the initiator, regardless of whether it is BnOH or Ph_2_CHOH, improves the control over the polymerization, leading to a polymer with a more controlled molecular weight and narrow polydispersity.

While the use of both BnOH and Ph_2_CHOH as initiators led to enhanced control over the polymerization and narrowed polydispersity, excessive concentrations of the alcohol initiator caused an alteration in the reaction pathway, resulting in unintended side reactions, particularly transesterification. In the case of using 100 equivalents of BnOH (Table 1, entry 8), significant quantities of benzyl lactate were observed, suggesting that transesterification became a dominant side reaction under these conditions. The occurrence of transesterification under high concentrations of the alcohol initiator is likely due to the increased availability of alcohol groups, which can act as nucleophiles, competing with the polymerization process and disrupting the desired reaction pathway. The formation of benzyl lactate in this case may also indicate that the reaction is no longer proceeding via a controlled mechanism but rather through an uncontrolled side reaction that introduces additional complexity into the polymerization. The dominance of transesterification in the presence of excess alcohol suggests that careful optimization of the initiator concentration is crucial to maintaining the integrity of the polymerization process. It highlights the need for a balance between sufficient initiator concentration to promote efficient polymerization and avoiding an excess that leads to side reactions such as transesterification.

**Figure 1:**
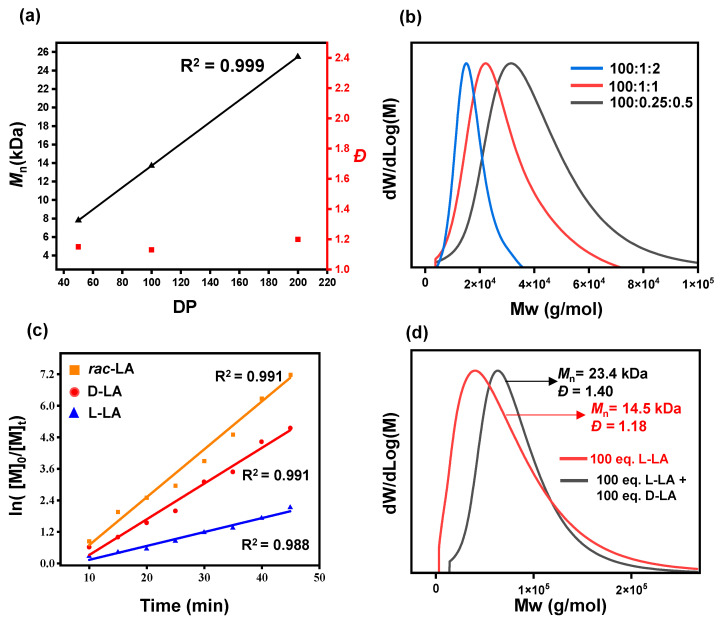
(**a**) Plots of different polymerization degrees (DPs) ([M]_0_/[I]_0_) versus *M*_n_ and (*Ð*) (Table 1, entries 2, 3, and 10). (**b**) Molecular weight (Mw) distribution of ROP of L-LA at different polymerization degrees (DPs) ([M]_0_/[I]_0_) versus *M*_n_ and (*Ð*) (Table 1, entries 2, 3, and 10). (**c**) Semilogarithmic plots of ln ([M]_0_/[M]_t_) versus time for LA. L-LA (blue squares, k_app_ = 0.0530 min^−1^, R_2_ = 0.988); D-LA (red circles, k_app_ = 0.1355 min^−1^, R_2_ = 0.991); *rac*-LA (orange triangles, k_app_ = 0.1764 min^−1^, R_2_ = 0.991) using LiHMDS. (**d**) Molecular weight (Mw) distribution of polymerization before and after the addition of 100 equiv. of D-lactide (Table 1, entry 13).

Lowering the catalyst concentration in the ring-opening polymerization of lactide demonstrated that efficient polymerization and good control could be achieved even under low catalyst loading. For example, at a monomer-to-catalyst-to-initiator ratio of 100:0.5:1, the reaction achieved a 60% conversion within 15 min, yielding a polymer with a molecular weight of 10.7 kDa and a narrow polydispersity (*Đ* = 1.09), showcasing the robust catalytic performance of LiHMDS (Table 1, entry 9). When the monomer-to-catalyst-to-initiator ratio was further increased to 100:0.25:0.5, complete polymer conversion was achieved within 30 min (Table 1, entry 10). This highlights the high activity of LiHMDS, even at very low concentrations, enabling efficient polymerization while maintaining excellent control over molecular weight distribution. Moreover, increasing the degree of polymerization (DP) under these conditions demonstrated a linear relationship between DP and molecular weight (Table 1, entries 2, 3, and 10). This linear increase in *M*_n_ as a function of DP is characteristic of living polymerization systems, wherein polymer chain growth proceeds uniformly without significant termination or transfer reactions. Kinetic analysis further confirmed the controlled nature of the polymerization, with the reaction kinetics following a first-order dependence on the monomer concentration, as reflected in the linear correlation of ln([M]_0_/[M]_t_) over time (Figure 1c). The stereochemistry of the monomer was found to significantly influence polymerization control. When LiHMDS was used for the ROP of D-lactide and *rac*-lactide, distinct differences in dispersity were observed (Table 1, entries 11 and 12). Specifically, the polymerization of *rac*-lactide resulted in a higher dispersity compared to D-lactide. This increased dispersity is likely attributable to the stereochemical heterogeneity of *rac*-lactide, which disrupts uniform chain propagation and introduces irregularities in the polymer microstructure. These findings underscore the critical influence of monomer stereochemistry on polymerization control and the sensitivity of LiHMDS to stereochemical variations during the ROP of lactide.

**Figure 2:**
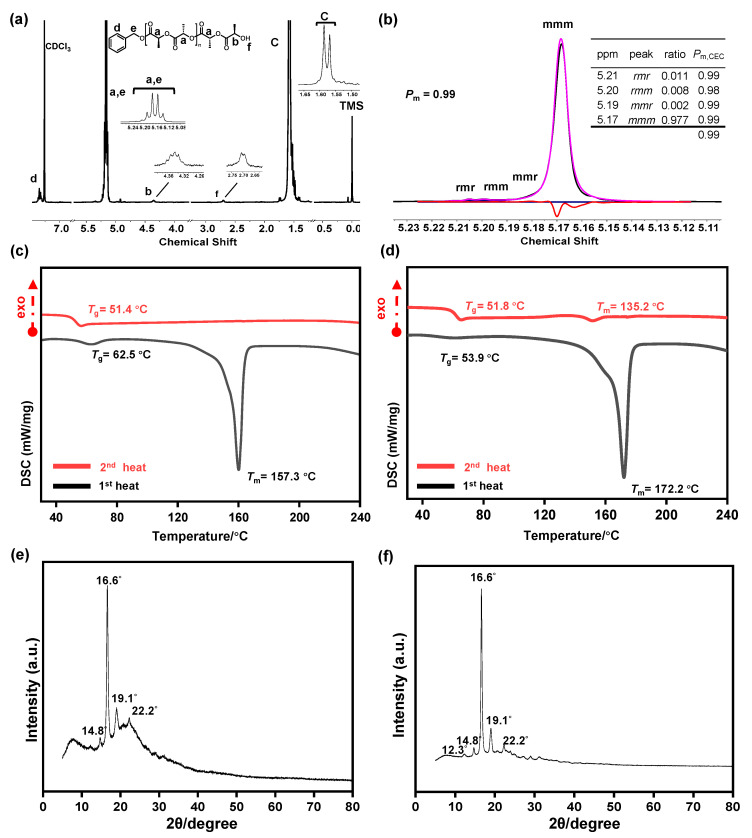
(**a**) ^1^H NMR of PLA after the addition of 100 equiv. of D-lactide. (**b**) Homonuclear decoupled ^1^H NMR spectra of PLA after the addition of 100 equiv. of D-lactide. (**c**) Differential scanning calorimetry (DSC) before the addition of 100 equiv. of D-lactide. (**d**) Differential scanning calorimetry (DSC) after the addition of 100 equiv. of D-lactide. (**e**) WAXD profile of PLA before the supernumerary addition of 100 equiv. of D-lactide. (**f**) WAXD profile of PLA after the supernumerary addition of 100 equiv. of D-lactide. Data correspond to Table 1, entry 13.

In our study, we also aimed to validate the concept of polymer chain extension by demonstrating the controlled addition of monomers during the ring-opening polymerization (ROP) of lactides. After completing the polymerization of 100 equivalents of L-lactide, an equal amount of D-lactide was introduced into the reaction system (Table 1, entry 13). This additional step led to a significant increase in molecular weight, as evidenced by the shift in the second molecular weight distribution curve of GPC (Figure 1d). This behavior is characteristic of living polymerization, where the active polymer chain ends remain available for further propagation, enabling precise chain extension without termination or side reactions. The stereochemical properties of the resulting polymers were analyzed to assess the catalyst’s ability to maintain control during the chain extension process. As shown in Figure 2a,b, the polymers synthesized after the addition of 100 equivalents of D-LA exhibited a high degree of isotacticity, with a probability of isotacticity (*P*_m_) up to 0.99. This high isotacticity indicates that the LiHMDS catalyst maintained excellent stereo-control throughout the polymerization process, even during the chain extension stage. Additionally, the lack of significant transesterification demonstrates the catalyst’s ability to preserve the stereochemical integrity of the polymer chains, which is critical for applications requiring precise structural and thermal properties.

Differential scanning calorimetry (DSC) analysis provided further insights into the thermal properties of the synthesized polylactide (PLA) samples before and after the addition of D-LA. Prior to the chain extension, the PLA showed a melting temperature (*T*_m_) of 157.3 °C and a glass transition temperature (*T*_g_) of 54.4 °C during the first heating cycle (Figure 2c). However, no melting peak was observed in the second heating cycle, suggesting that the thermal properties were not retained upon reheating. After the addition of D-LA, the second heating cycle revealed a *T*_m_ of approximately 135.2 °C (Figure 2d), with no significant change in *T*_g_. This observation in thermal behavior confirms the formation of stereocomplex structures in the modified PLA, resulting from the interaction between the enantiomeric lactide units. Wide angle X-ray diffraction (WAXD) analysis further supported these findings. The pattern of PLA obtained solely from L-LA exhibited broad diffraction peaks as shown in (Figure 2e). However, after the addition of D-LA and the formation of stereocomplex PLA, the diffraction peaks became significantly sharper and more intense (Figure 2f). This confirms the formation of a more crystalline polymer due to the stereocomplexation between L- and D-lactide units. The enhanced crystallinity of the modified PLA correlates with its improved thermal stability and mechanical properties, which are desirable for various high-performance applications.

To further evaluate the catalytic efficiency of LiHMDS and its versatility across different monomer systems, the catalytic system was extended to include the ring-opening polymerization of δ-valerolactone (δ-VL) and ε-caprolactone (ε-CL), as shown in Table 2 (entries 1 to 4). Both δ-VL and ε-CL are cyclic esters that have garnered considerable interest due to their ability to produce biodegradable and biocompatible polyesters, making them ideal candidates for applications in biomedical devices, sustainable packaging solutions, and other environmentally friendly products [30].

**Figure 3:**
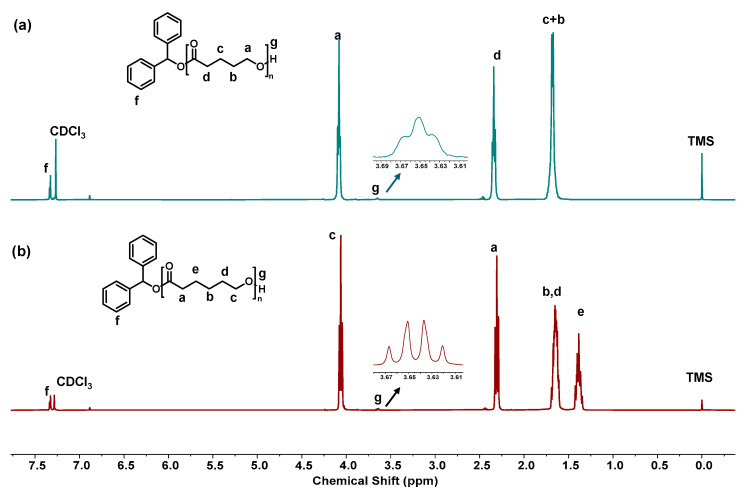
^1^H NMR spectrum for (**a**) poly(δ-VL) obtained by LiHMDS (Table 2, entry 2) and (**b**) poly(ε -CL) obtained by LiHMDS (Table 2, entry 4).

In entries 1 and 2, δ-VL was polymerized under different reaction conditions. In entry 1, the molar ratio of [M]_0_/[Cat]_0_/[OH]_0_ was 100:0.25:1, and the reaction time was three minutes. The LiHMDS catalyst demonstrated remarkable efficiency, achieving nearly 99% monomer conversion in just three minutes. However, in the polymerization studies using a monomer-to-initiator-to-catalyst ratio of 100:0.25:0.5, the ^1^H NMR spectra (Figures S3 and S5) did not exhibit distinct aromatic proton signals of the initiator, likely due to their low concentration relative to the polymer backbone. To improve the detectability of these aromatic protons, we adjusted the reaction ratio to 50:1:2 (Table 2, entry 2). Under these conditions, the increased concentration of the aromatic initiator resulted in clear and distinct aromatic proton signals in the ^1^H NMR spectra, as shown below (Figure 3a,b).

**Figure 4:**
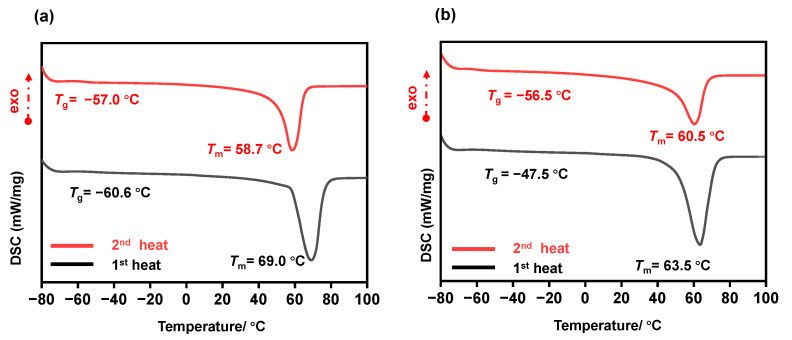
Differential scanning calorimetry (DSC) for (**a**) PVL obtained by LiHMDS and for (**b**) PCL obtained by LiHMDS.

Moving to entries 3 and 4, ε-CL was polymerized under similar reaction conditions. In entry 3, the molar ratio of [M]_0_/[Cat]_0_/[OH]_0_ was again 100:0.25:1, and the reaction time was three minutes. As in entry 1 with δ-VL, near-complete conversion (99%) was achieved. In entry 4, the molar ratio was adjusted to 50:1:2, and the reaction time was again shortened to two minutes. Similar to entry 2, this change resulted in near-complete conversion (99%) but led to a higher dispersity index (*Đ* = 1.53), indicating that the polymerization was less controlled and produced a broader range of polymer chain lengths. The faster reaction time and altered catalyst-to-monomer ratio likely limited the ability of LiHMDS to achieve precise control over the polymerization, leading to a broader distribution of polymer sizes. Thermal analysis of the resulting homopolymers was conducted using differential scanning calorimetry (DSC), which provided insight into the thermal properties of the polymers (Figure 4a,b). During the second heating cycle, poly(δ-VL) (PVL) exhibited a melting temperature (*T*_m_) of 58.7 °C, while poly(ε-CL) (PCL) displayed a slightly higher *T*_m_ of 60.5 °C. The proximity of these melting temperatures indicates that both polymers exhibit similar crystalline behaviors, which is consistent with the expected properties of semi-crystalline polymers derived from lactone monomers like δ-VL and ε-CL.

## 4. Conclusions

In summary, the highly active catalyst lithium bis(trimethylsilyl)amide (LiHMDS) was used to catalyze the ring-opening polymerization of various cyclic esters at room temperature. This catalyst demonstrated rapid and efficient polymerization of lactide even at elevated monomer concentrations, achieving controlled molecular weights and narrow polydispersities. It effectively converted a range of cyclic esters, including δ-valerolactone and ε-caprolactone, into polyesters. Notably, the addition of an initiator significantly enhanced the catalytic activity, resulting in polyesters with narrower molecular weight distributions.

## Figures and Tables

**Table 1:**
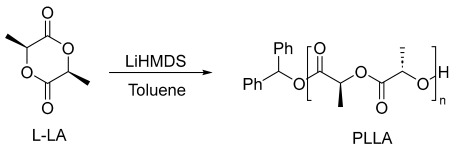
Ring-opening polymerization of L-Lactide catalyzed by LiHMDS ^1^.

Entry	[M]_0_/[Cat]_0_/[OH]_0_ ^2^	Initiator	Time	Conv. ^3^ (%)	*M*_n,calcd_ ^4^ (kDa)	*M*_n,GPC_ ^5^ (kDa)	*Ð* ^5^
1	100:1:0	-	12 h	98	-	25.3	1.55
2	100:1:1	BnOH	30 min	92	13.3	13.7	1.15
3	100:1:2	BnOH	8 min	93	6.8	7.8	1.13
4	100:1:4	BnOH	3 min	90	3.3	3.8	1.10
5	100:1:1	Ph_2_CHOH	30 min	90	13.2	16.4	1.12
6	100:1:2	Ph_2_CHOH	10 min	92	6.7	13.9	1.18
7	100:1:4	Ph_2_CHOH	5 min	91	3.3	3.9	1.08
8 ^6^	100:1:400	BnOH	3 min	99	-	-	-
9	100:0.5:1	BnOH	15 min	60	8.7	10.7	1.09
10	100:0.25:0.5	BnOH	30 min	92	26.6	25.1	1.20
11 ^7^	100:0.25:0.5	BnOH	25 min	80	23.12	25.1	1.20
12 ^8^	100:0.25:0.5	BnOH	14 min	75	21.71	22.3	1.47
13 ^9^	100 + 100:1:2	BnOH	8 + 20 min	94	13.5	23.4	1.40

^1^ Carried out at ambient temperature (25 ± 5 °C) unless indicated otherwise, using BnOH as initiator, [L-LA]_0_ = 1.0 M. ^2^ M (monomer), Cat (catalyst), and OH (hydroxyl initiator) ratios. ^3^ The conversion was determined by ^1^H NMR spectroscopy by calculating the integral ratio between the methine regions of lactide and PLA. ^4^ *M*_n_,_calcd_ =([L-LA]_0_/[OH]_0_) × 144.14 × conv. (%) + *M*_n,initiator_. ^5^ Apparent number-average molar mass (*M*_n_) and dispersity (*Ð*) values were determined by GPC in THF using polystyrene standards for calibration and corrected using the factor 0.58 for polylactide. ^6^ Benzyl alcohol was used as the solvent and the product was benzyl lactate. ^7^ For [D-LA]_0_= 1.0 M and ^8^ [*rac*-LA]_0_ = 1.0 M. ^9^ Here, 100 equiv. of D-LA was supplemented after the polymerization of 100 equiv. of L-LA.

**Table 2:**
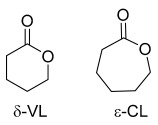
Result for ROP of different lactones ^1^.

Entry	M	[M]_0_/[Cat]_0_/[OH]_0_^2^	Time (min)	Conv. (%) ^3^	*M*_n,calcd_ (kDa) ^4^	*M_n,GPC_* (kDa) ^5^	*Ð* ^5^
1	VL	100:0.25:1	3	99	10.0	9.9	1.35
2	VL	50:1:2	2	99	2.7	6.2	1.61
3	CL	100:0.25:1	3	99	11.5	15.6	1.22
4	CL	50:1:2	2	99	3.0	7.7	1.54

^1^ The polymerizations were carried out with [M]_0_ = 1.0 M in toluene [M]_0_/[Li]_0_/[Ph_2_CHOH]_0_ = 100:0.25:1. ^2^ M (monomer), Cat (catalyst), and OH (hydroxyl initiator) ratios. ^3^ Monomer conversion was determined and calculated by the ^1^H NMR spectrum of the crude product in CDCl_3_. ^4^ Theoretical number-average molar mass (*M*_n_,_calcd_) was calculated from [M]_0_/[I]_0_ and monomer conversion. ^5^ Apparent number-average molar mass (*M*_n_,_GPC_) and dispersity (*Ð*) value were determined by GPC in THF using polystyrene standards for calibration and corrected using the factor 0.57 for δ-VL and 0.56 for ε-CL.

## Data Availability

Data are contained within the article or Supplementary Material.

## Supplementary Materials

The following supporting information can be downloaded at: https://www.mdpi.com/article/10.3390/polym17030429/s1, Figure S1:1H NMR spectrum of PLLA obtained by LiHMDS (Table 1, entry 2) (400 MHz, Chloroform-d, 298 K); Figure S2. 13C NMR spectrum of PLLA obtained by LiHMDS (Table 1, entry 2) (400 MHz, Chloroform-d, 298 K); Figure S3. 1H NMR spectrum of PVL obtained by LiHMDS (Table 2, entry 1) (400 MHz, Chloroform-d, 298 K); Figure S4. 13C NMR spectrum of PVL obtained by LiHMDS (Table 2, entry 1) (400 MHz, Chloroform-d, 298 K); Figure S5. 1H NMR spectrum of PCL obtained by LiHMDS (Table 2, entry 2) (400 MHz, Chloroform-d, 298 K); Figure S6. 13C NMR spectrum of PCL obtained by LiHMDS (Table 2, entry 2) (400 MHz, Chloroform-d, 298 K); Figure S7. GPC trace for PLLA obtained by LiHMDS (Table 1, entry 1); Figure S8. GPC trace for PLLA obtained by LiHMDS (Table 1, entry 2); Figure S9. GPC trace for PLLA obtained by LiHMDS (Table 1, entry 3); Figure S10. GPC trace for PLLA obtained by LiHMDS (Table 1, entry 4); Figure S11. GPC trace for PLLA obtained by LiHMDS (Table 1, entry 5); Figure S12. GPC trace for PLLA obtained by LiHMDS (Table 1, entry 6); Figure S13. GPC trace for PLLA obtained by LiHMDS (Table 1, entry 7); Figure S14. GPC trace for PLLA obtained by LiHMDS (Table 1, entry 9); Figure S15. GPC trace for PLLA obtained by LiHMDS (Table 1, entry 10); Figure S16. GPC trace for PLLA obtained by LiHMDS (Table 1, entry 11); Figure S17. GPC trace for PLLA obtained by LiHMDS (Table 1, entry 12); Figure S18. GPC trace for PLA obtained by LiHMDS (Table 1, entry 13); Figure S19. GPC trace for PVL obtained by LiHMDS (Table 2, entry 1); Figure S20. GPC trace for PVL obtained by LiHMDS (Table 2, entry 2); Figure S21. GPC trace for PCL obtained by LiHMDS (Table 2, entry 3); Figure S22. GPC trace for PCL obtained by LiHMDS (Table 2, entry 4); Figure S23. Differential scanning calorimetry (DSC) before the supernumerary addition of 100 equiv. of D-lactide (Table 1, entry 13); Figure S24. Differential scanning calorimetry (DSC) after the supernumerary addition of 100 equiv. of D-lactide (Table 1, entry 13); Figure S25. Differential scanning calorimetry (DSC) for PVL obtained by LiHMDS (Table 2, entry 1); Figure S26. Differential scanning calorimetry (DSC) for PCL obtained by LiHMDS (Table 2, entry 3); Figure S27. Proposed reaction mechanism for PLLA by LiHMDS.

## Abbreviations

Name	Abbreviations
Differential scanning calorimetry	DSC
Nuclear magnetic resonance	NMR
Gel permeation chromatography	GPC
Melting temperature	*T*_m_
Glass transition temperature	*T_g_*
Probability of racemic diad	*P*_m_
Chain-end control mechanism	CEC
Number-average molecular weight	*M*_n_
Polydispersity	*Ð*
Monomer	M
Catalyst	Cat
Hydroxyl initiator	OH
