# Supplementary material for: Exploring the Catalytic Efficiency of Lithium Bis(trimethylsilyl)amide (LiHMDS) in Lactide Polymerization
Source: Polymers (Basel). 2025 Feb 6;17(3):429. doi: 10.3390/polym17030429 (PMC11821231; doi:10.3390/polym17030429)
Supplement: Supplementary file 1 [file polymers-17-00429-s001.zip › polymers-3389888-supplementary.pdf]

Supplementary Material

Exploring the Catalytic Efficiency of lithium bis(trimethylsilyl)amide (LiHMDS) in Lactide Polymerization

Almas Kiran,[1,2] Achukee Chinedu Kingsley, [1,2] and Hassan Ahmed [2,3,*]

[1] University of Chinese Academy of Science, Beijing, 100049, China.
[2] Key Laboratory of Photoelectric Conversion and Utilization of Solar Energy, Qingdao Institute of Bioenergy and Bioprocess Technology, Chinese Academy of Sciences, Qingdao, 266101, China
[3] Shandong Energy Institute, Qingdao, 266101, China
* Correspondence: hassan@qibebt.ac.cn

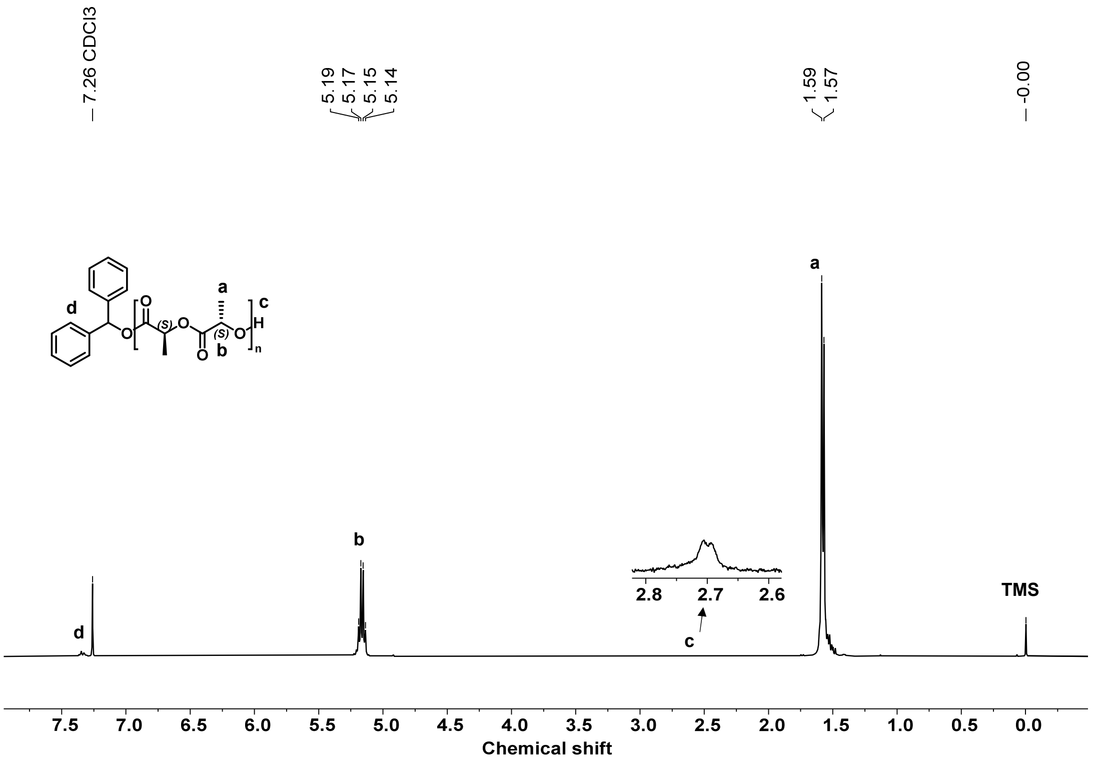

Figure S1. ^{1}H NMR spectrum of PLLA obtained by LiHMDS (Table 1, entry 2) (400 MHz, Chloroform-*d*, 298 K).

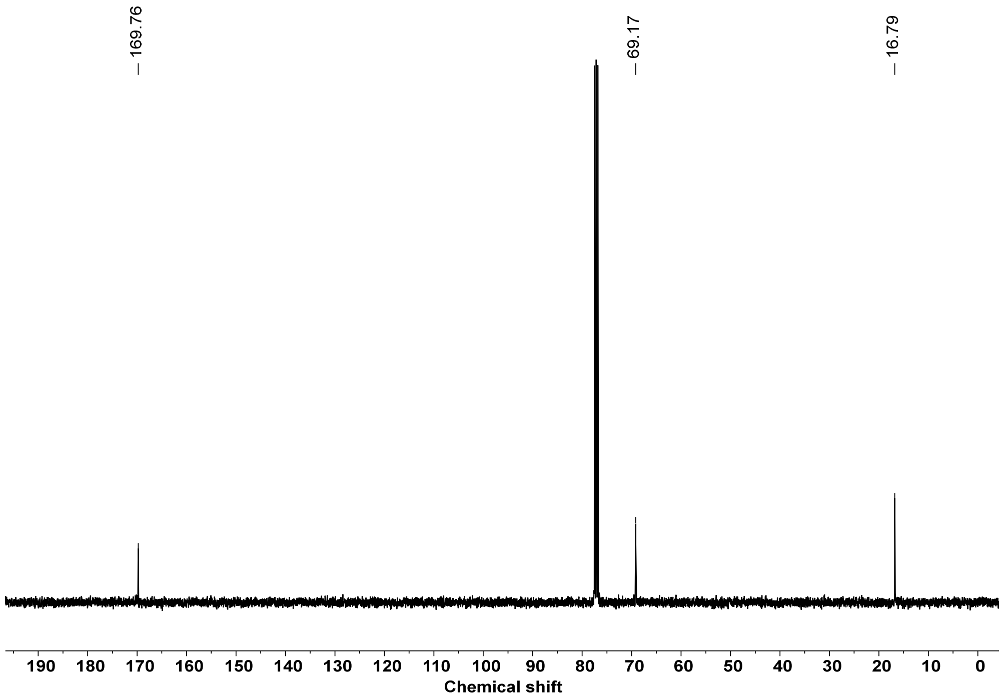

Figure S2. ^{13}C NMR spectrum of PLLA obtained by LiHMDS (Table 1, entry 2) (400 MHz, Chloroform-*d*, 298 K).

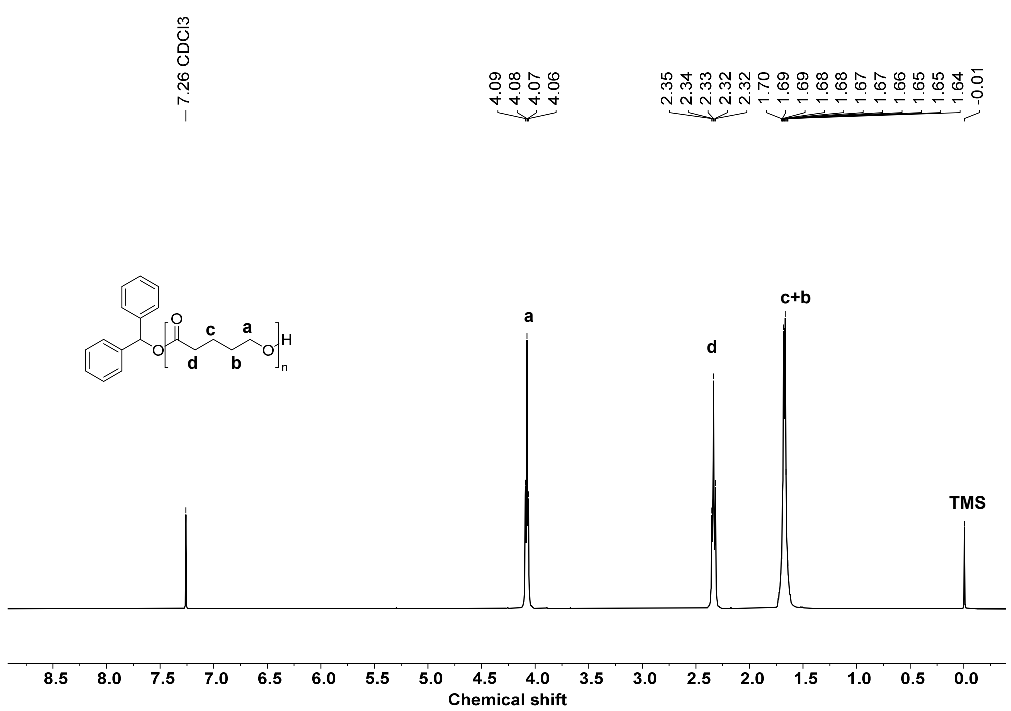

Figure S3. ^{1}H NMR spectrum of PVL obtained by LiHMDS (Table 2, entry 1) (400 MHz, Chloroform-*d*, 298 K).

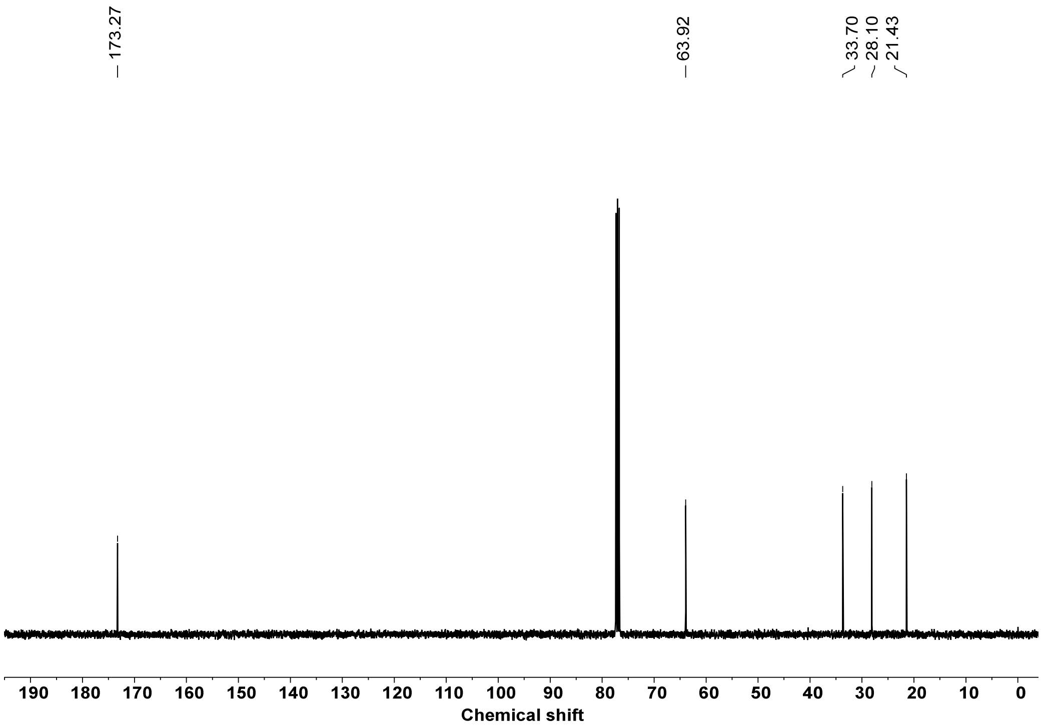

Figure S4. ^{13}C NMR spectrum of PVL obtained by LiHMDS (Table 2, entry 1) (400 MHz, Chloroform-*d*, 298 K).

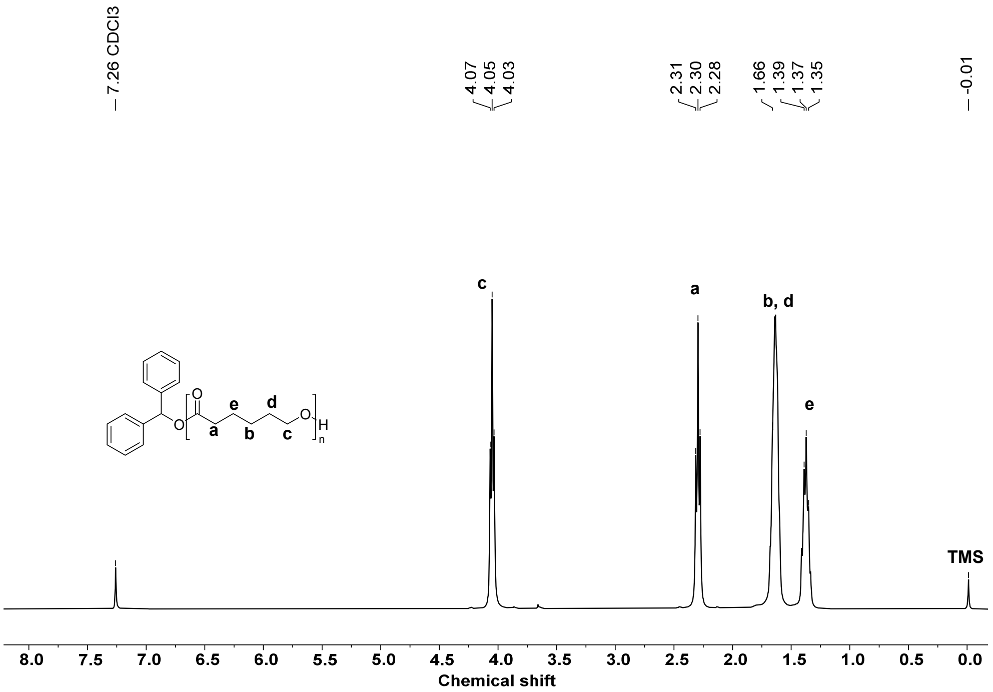

Figure S5. ^{1}H NMR spectrum of PCL obtained by LiHMDS (Table 2, entry 2) (400 MHz, Chloroform-*d*, 298 K).

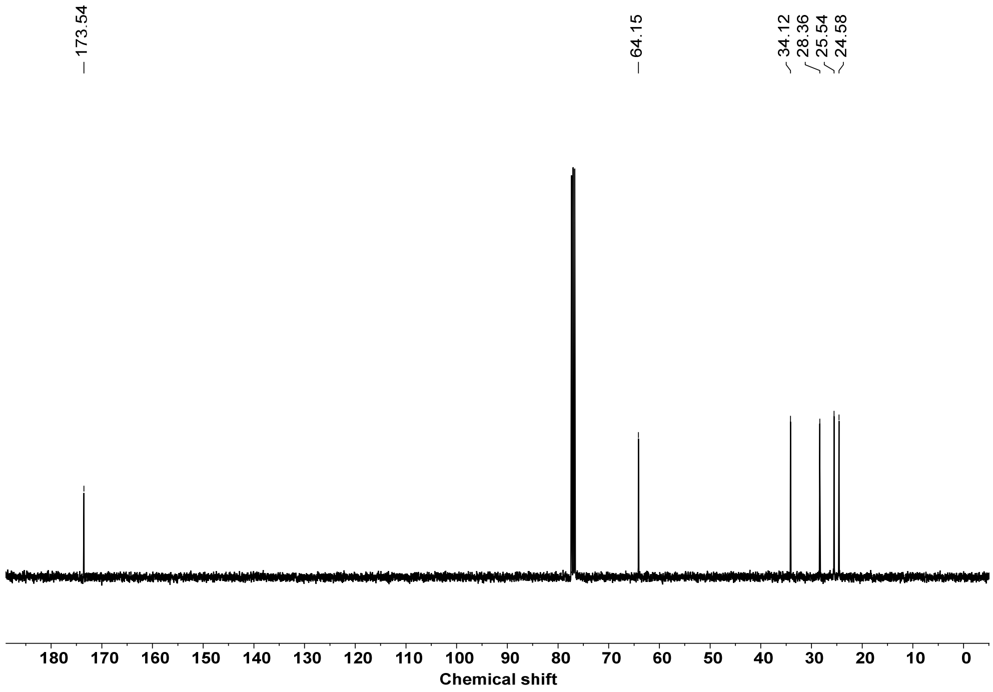

Figure S6. ^{13}C NMR spectrum of PCL obtained by LiHMDS (Table 2, entry 2) (400 MHz, Chloroform-*d*, 298 K).

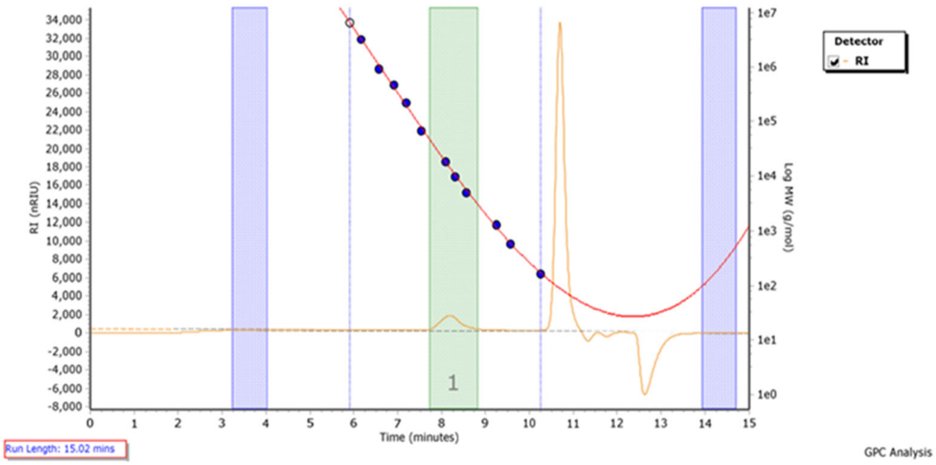

Molecular Weight Averages

Peak	Mp (g/mol)	Mn (g/mol)	Mw (g/mol)	Mz (g/mol)	Mz+1 (g/mol)	Mv (g/mol)	PD
Peak 1	64953	43670	67669	95599	120127	91741	1.55

Figure S7. GPC trace for PLLA obtained by LiHMDS (Table 1, entry 1).

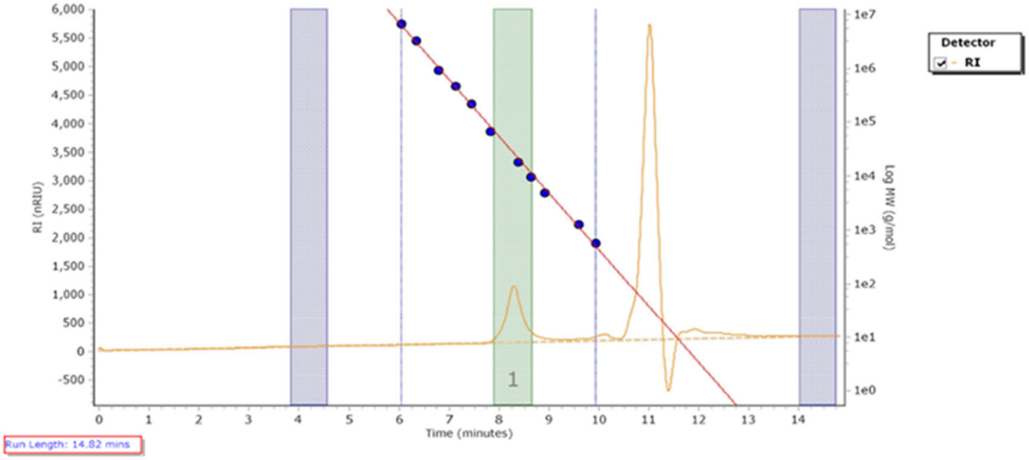

Molecular Weight Averages

Peak	Mp (g/mol)	Mn (g/mol)	Mw (g/mol)	Mz (g/mol)	Mz+1 (g/mol)	Mv (g/mol)
Peak 1	25705	23571	27296	31508	36080	26705

PD
1.158

Figure S8. GPC trace for PLLA obtained by LiHMDS (Table 1, entry 2).

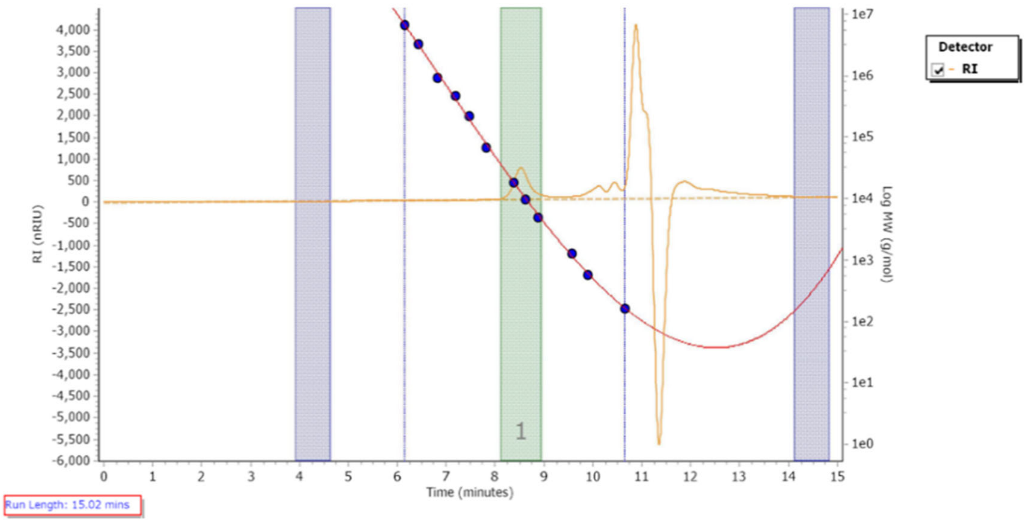

Molecular Weight Averages

Peak	Mp (g/mol)	Mn (g/mol)	Mw (g/mol)	Mz (g/mol)	Mz+1 (g/mol)	Mv (g/mol)
Peak 1	14962	13483	15258	17039	18826	14995

PD
1.132

Figure S9. GPC trace for PLLA obtained by LiHMDS (Table 1, entry 3).

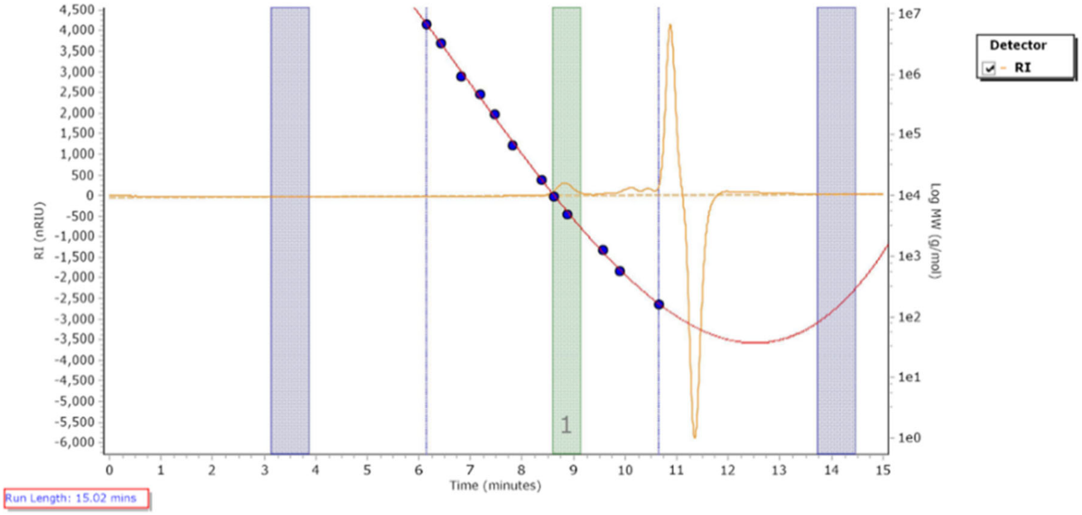

Molecular Weight Averages

Peak	Mp (g/mol)	Mn (g/mol)	Mw (g/mol)	Mz (g/mol)	Mz+1 (g/mol)	Mv (g/mol)
Peak 1	6941	6573	7248	7955	8663	7145

PD
1.103

Figure S10. GPC trace for PLLA obtained by LiHMDS (Table 1, entry 4).

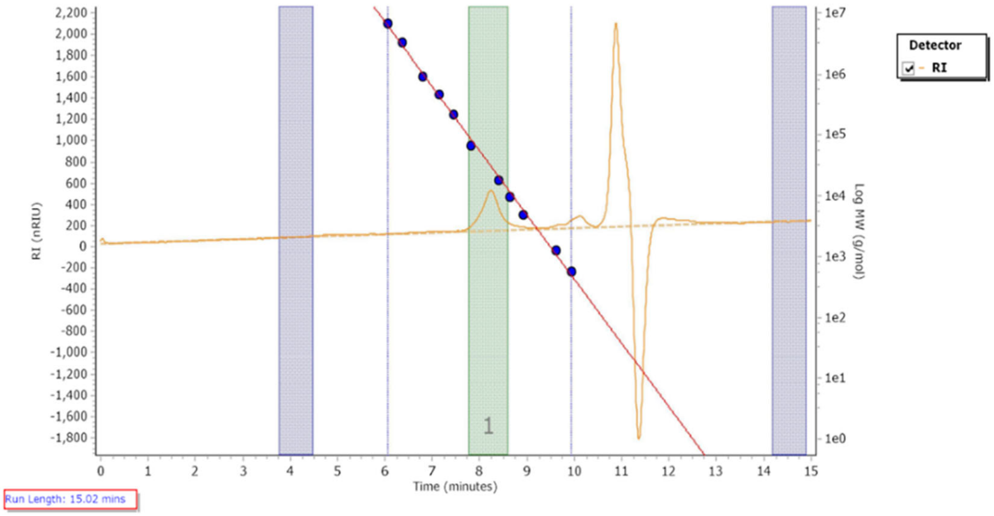

Molecular Weight Averages

Peak	Mp (g/mol)	Mn (g/mol)	Mw (g/mol)	Mz (g/mol)	Mz+1 (g/mol)	Mv (g/mol)
Peak 1	30079	28330	34144	41209	48971	33183

PD
1.205

Figure S11. GPC trace for PLLA obtained by LiHMDS (Table 1, entry 5).

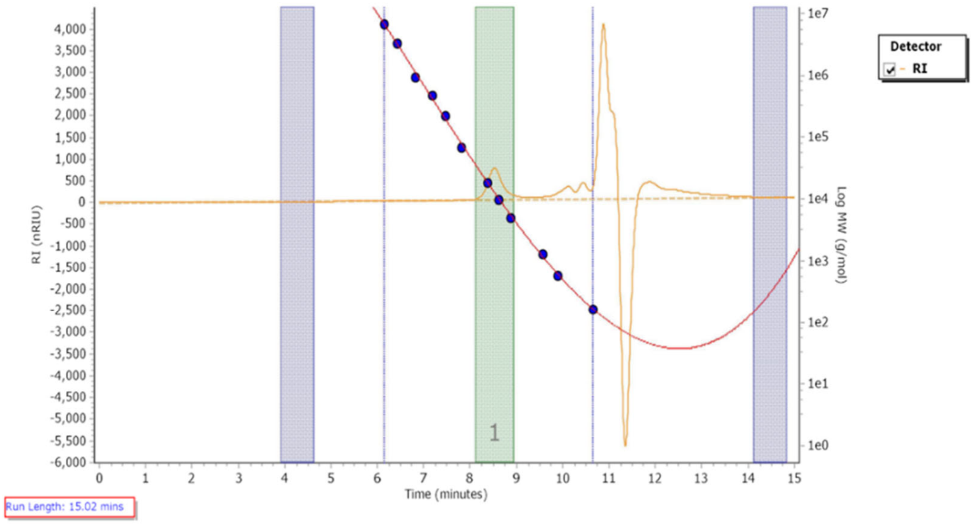

Molecular Weight Averages

Peak	Mp (g/mol)	Mn (g/mol)	Mw (g/mol)	Mz (g/mol)	Mz+1 (g/mol)	Mv (g/mol)
Peak 1	25260	23996	28316	33335	38741	27619

PD
1.18

Figure S12. GPC trace for PLLA obtained by LiHMDS (Table 1, entry 6).

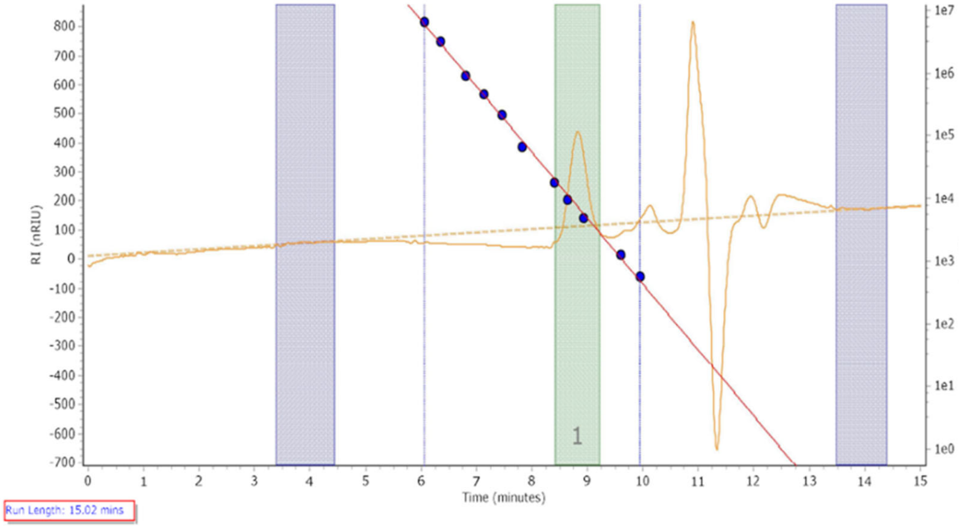

Molecular Weight Averages

Peak	Mp (g/mol)	Mn (g/mol)	Mw (g/mol)	Mz (g/mol)	Mz+1 (g/mol)	Mv (g/mol)
Peak 1	7314	6866	7444	8037	8619	7356

PD
1.084

Figure S13. GPC trace for PLLA obtained by LiHMDS (Table 1, entry 7).

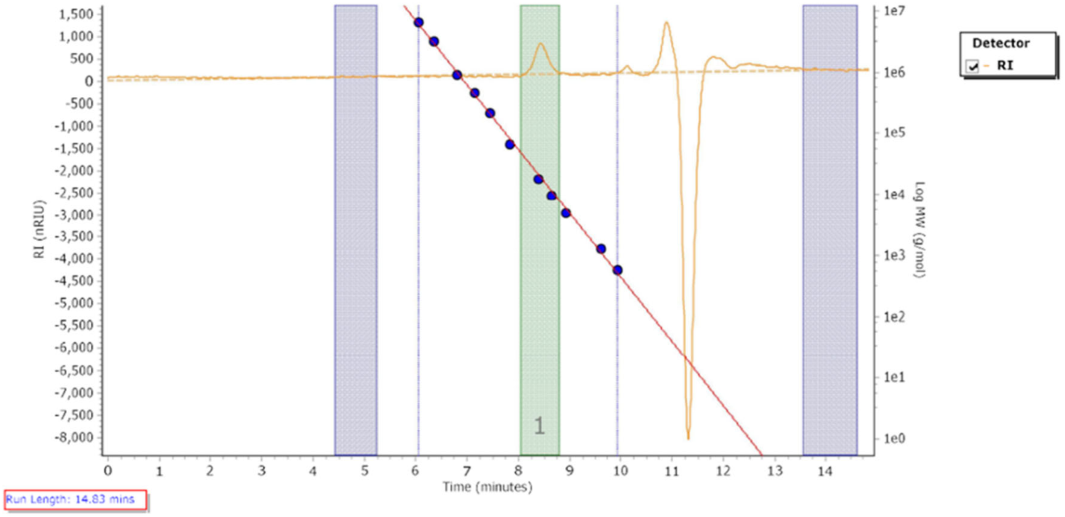

Molecular Weight Averages

Peak	Mp (g/mol)	Mn (g/mol)	Mw (g/mol)	Mz (g/mol)	Mz+1 (g/mol)	Mv (g/mol)
Peak 1	18449	17290	18919	20581	22251	18675

PD
1.094

Figure S14. GPC trace for PLLA obtained by LiHMDS (Table 1, entry 9).

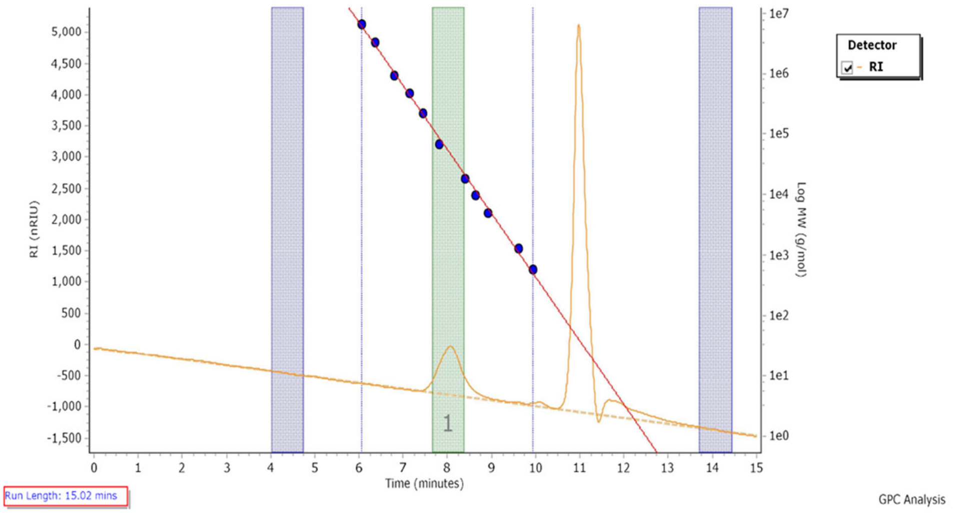

Molecular Weight Averages

Peak	Mp (g/mol)	Mn (g/mol)	Mw (g/mol)	Mz (g/mol)	Mz+1 (g/mol)	Mv (g/mol)
Peak 1	44162	43164	52143	62534	72754	50676

PD
1.208

Figure S15. GPC trace for PLLA obtained by LiHMDS (Table 1, entry 10).

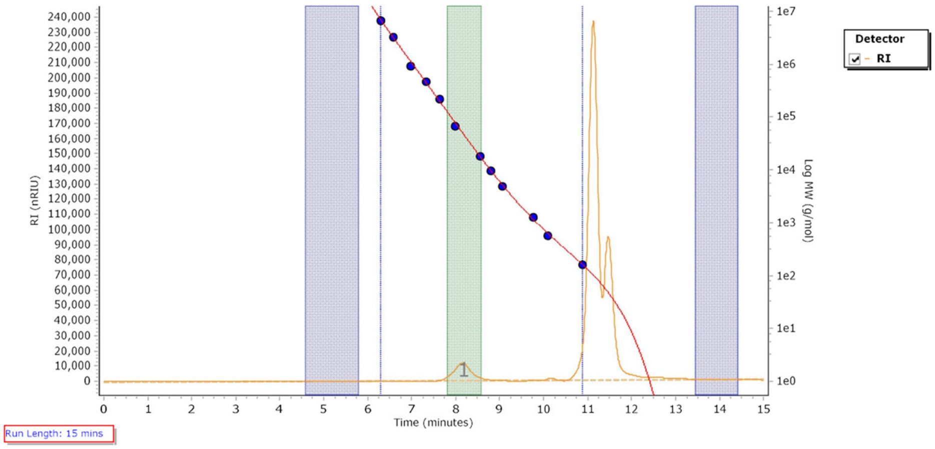

Molecular Weight Averages

Peak	Mp (g/mol)	Mn (g/mol)	Mw (g/mol)	Mz (g/mol)	Mz+1 (g/mol)	Mv (g/mol)
Peak 1	47990	43609	53386	63929	74108	51866

PD
1.224

Figure S16. GPC trace for PLLA obtained by LiHMDS (Table 1, entry 11).

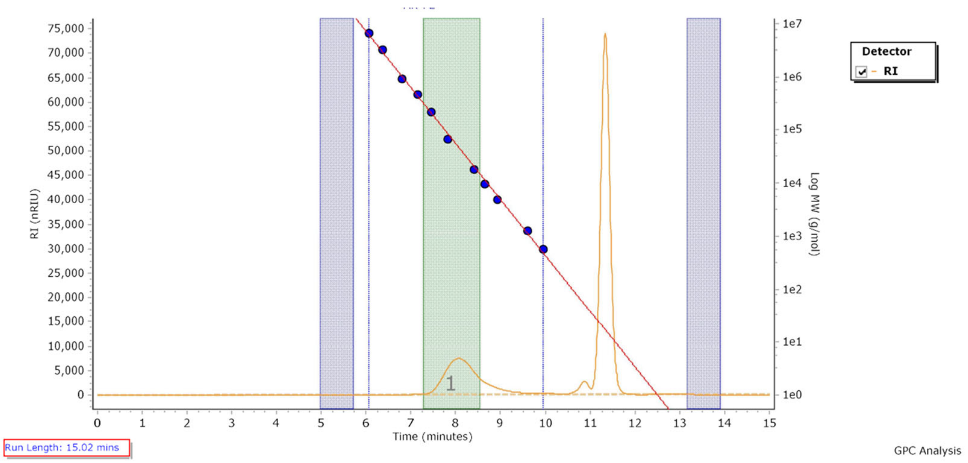

Molecular Weight Averages

Peak	Mp (g/mol)	Mn (g/mol)	Mw (g/mol)	Mz (g/mol)	Mz+1 (g/mol)	Mv (g/mol)
Peak 1	46537	38449	56681	83573	115656	53346

PD
1.474

Figure S17. GPC trace for PLLA obtained by LiHMDS (Table 1, entry 12).

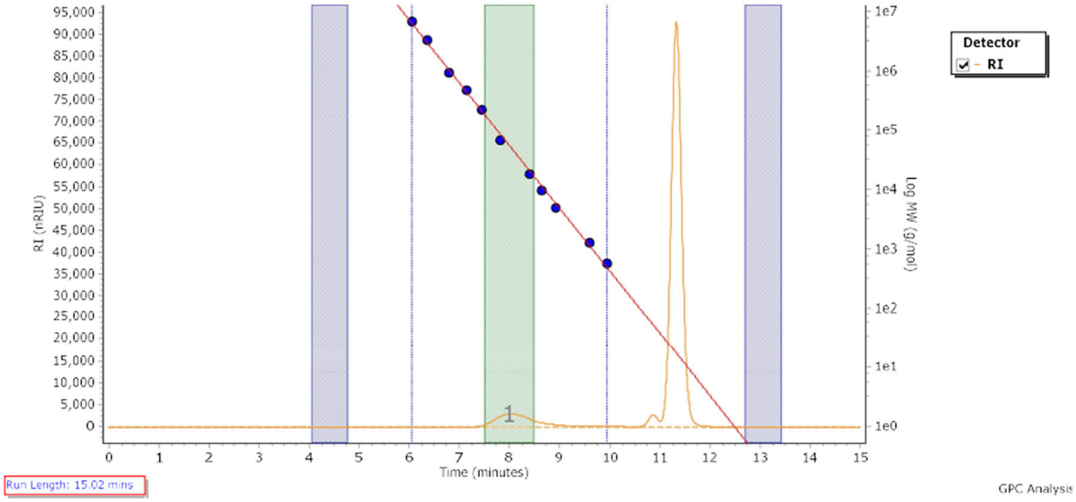

Molecular Weight Averages

Peak	Mp (g/mol)	Mn (g/mol)	Mw (g/mol)	Mz (g/mol)	Mz+1 (g/mol)	Mv (g/mol)
Peak 1	49039	40387	56524	76661	95997	53772

PD
1.4

Figure S18. GPC trace for PLA obtained by LiHMDS (Table 1, entry 13).

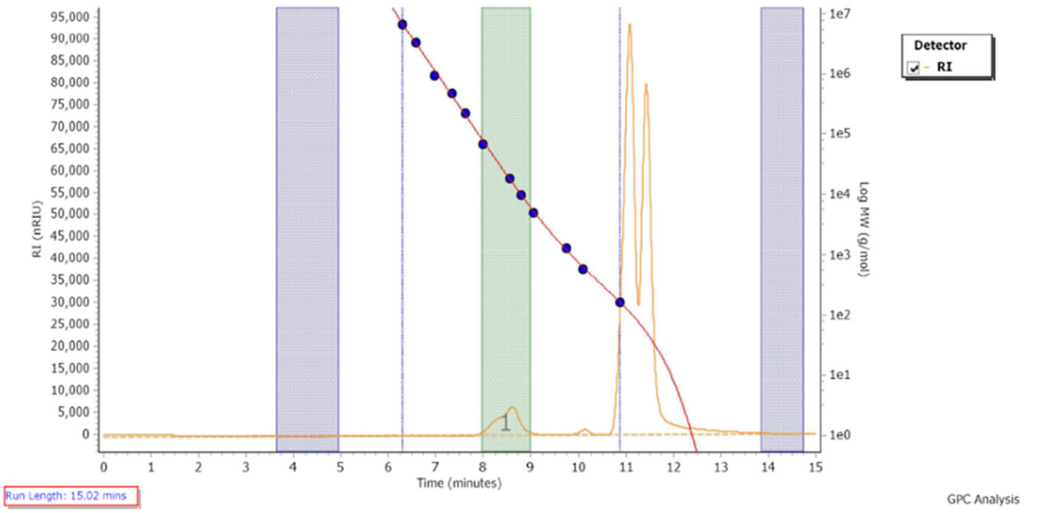

Molecular Weight Averages

Peak	Mp (g/mol)	Mn (g/mol)	Mw (g/mol)	Mz (g/mol)	Mz+1 (g/mol)	Mv (g/mol)
Peak 1	15223	17364	23602	32297	41649	22481

PD
1.359

Figure S19. GPC trace for PVL obtained by LiHMDS (Table 2, entry 1).

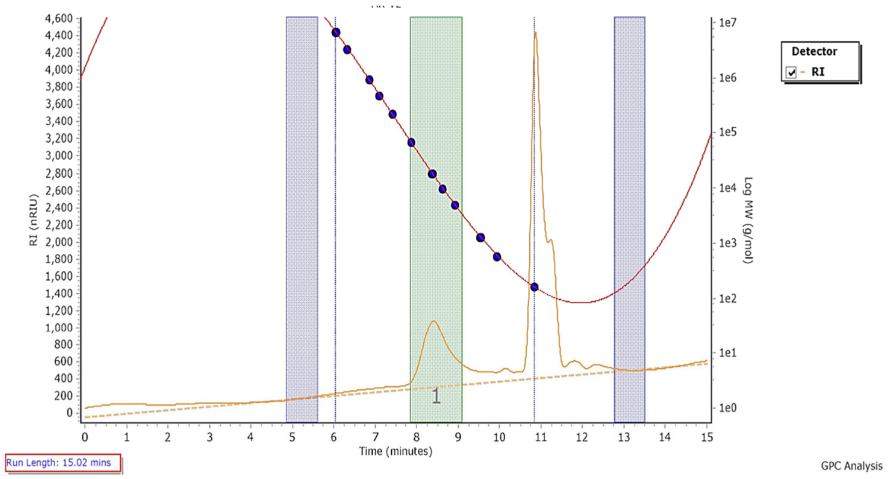

Molecular Weight Averages

Peak	Mp (g/mol)	Mn (g/mol)	Mw (g/mol)	Mz (g/mol)	Mz+1 (g/mol)	Mv (g/mol)
Peak 1	16905	10889	17485	25793	33908	16367

PD
1.606

Figure S20. GPC trace for PVL obtained by LiHMDS (Table 2, entry 2).

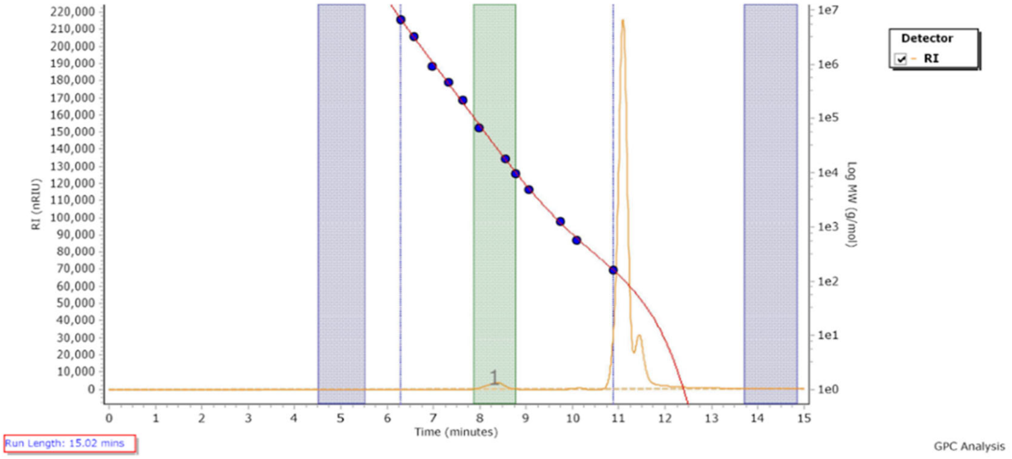

Molecular Weight Averages

Peak	Mp (g/mol)	Mn (g/mol)	Mw (g/mol)	Mz (g/mol)	Mz+1 (g/mol)	Mv (g/mol)
Peak 1	26869	27996	34262	42119	50832	33208
**PD**						
1.224						

Figure S21. GPC trace for PCL obtained by LiHMDS (Table 2, entry 3).

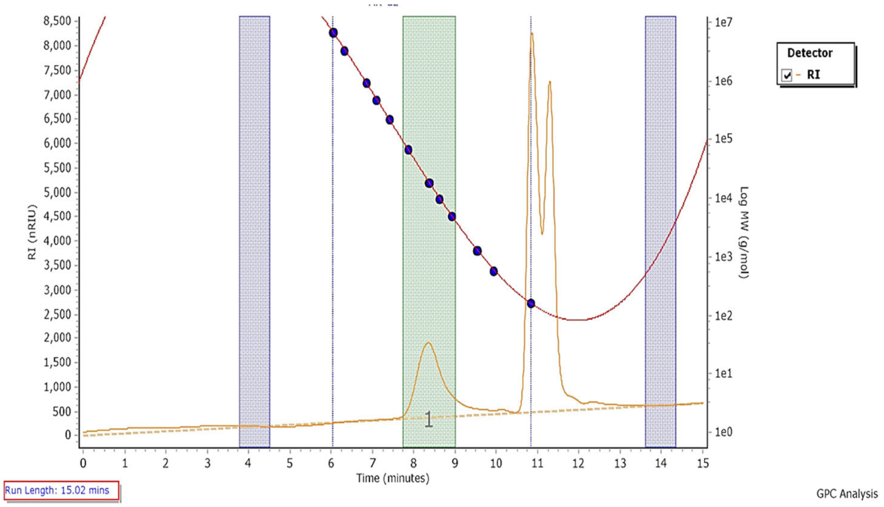

Molecular Weight Averages

Peak	Mp (g/mol)	Mn (g/mol)	Mw (g/mol)	Mz (g/mol)	Mz+1 (g/mol)	Mv (g/mol)
Peak 1	19497	13814	21198	30494	40174	19959
**PD**						
1.535						

Figure S22. GPC trace for PCL obtained by LiHMDS (Table 2, entry 4).

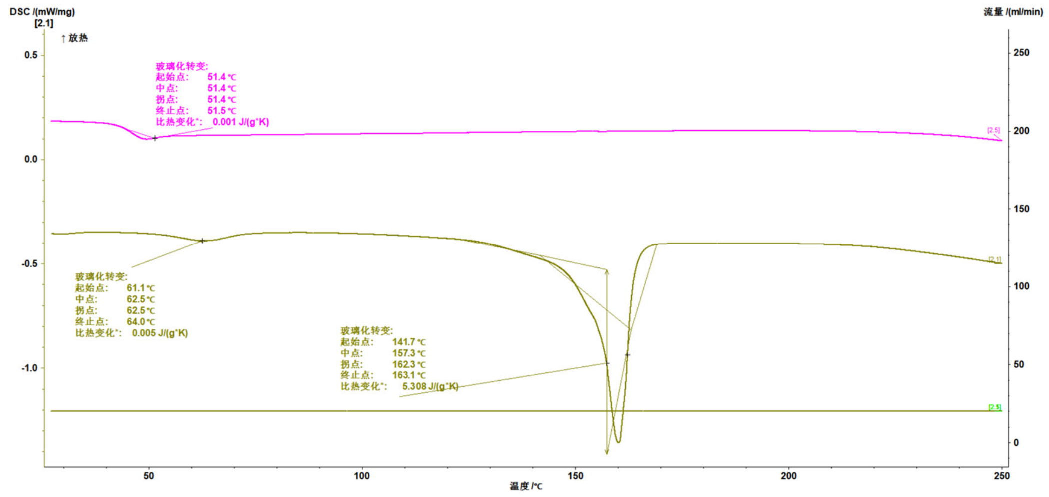

Figure S23. Differential scanning calorimetry (DSC) before the supernumerary addition of 100 equiv. of D-lactide (Table 1, entry 13).

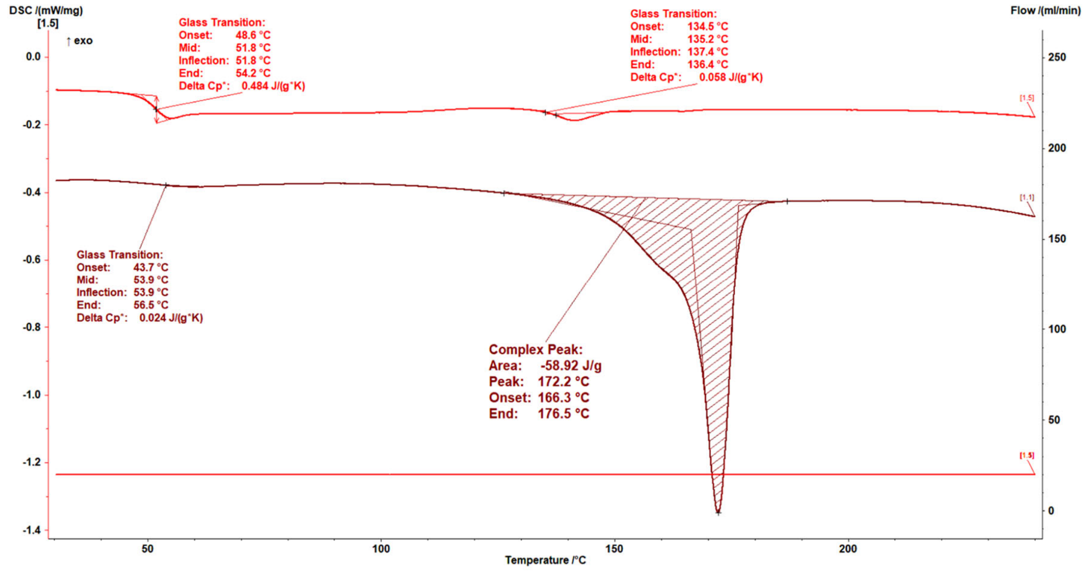

Figure S24. Differential scanning calorimetry (DSC) after the supernumerary addition of 100 equiv. of D-lactide (Table 1, entry 13).

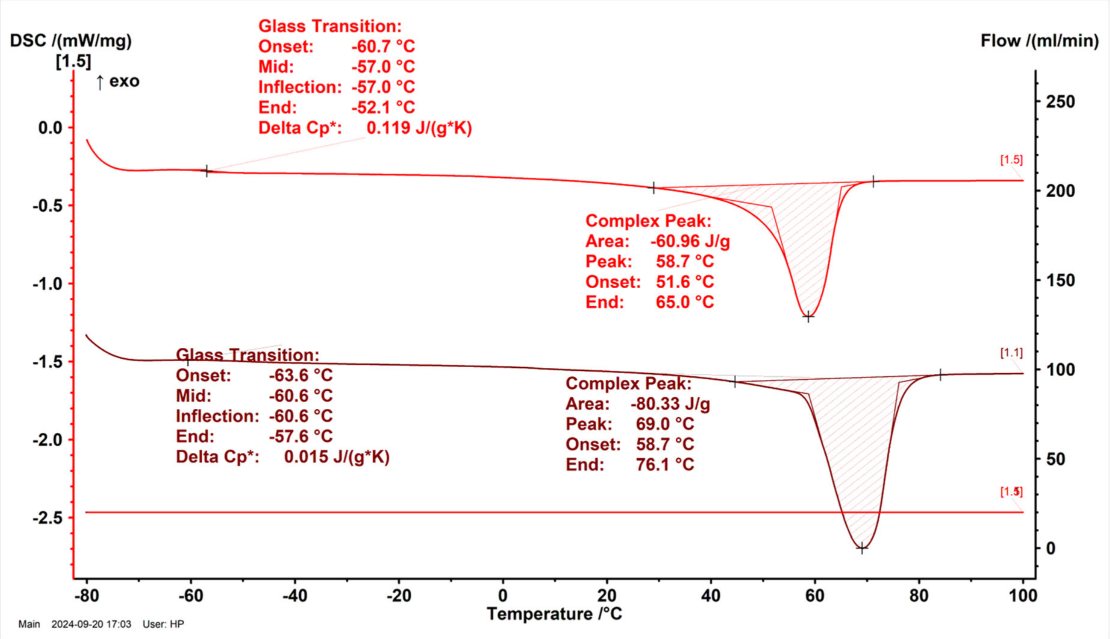

Figure S25. Differential scanning calorimetry (DSC) for PVL obtained by LiHMDS (Table 2, entry 1).

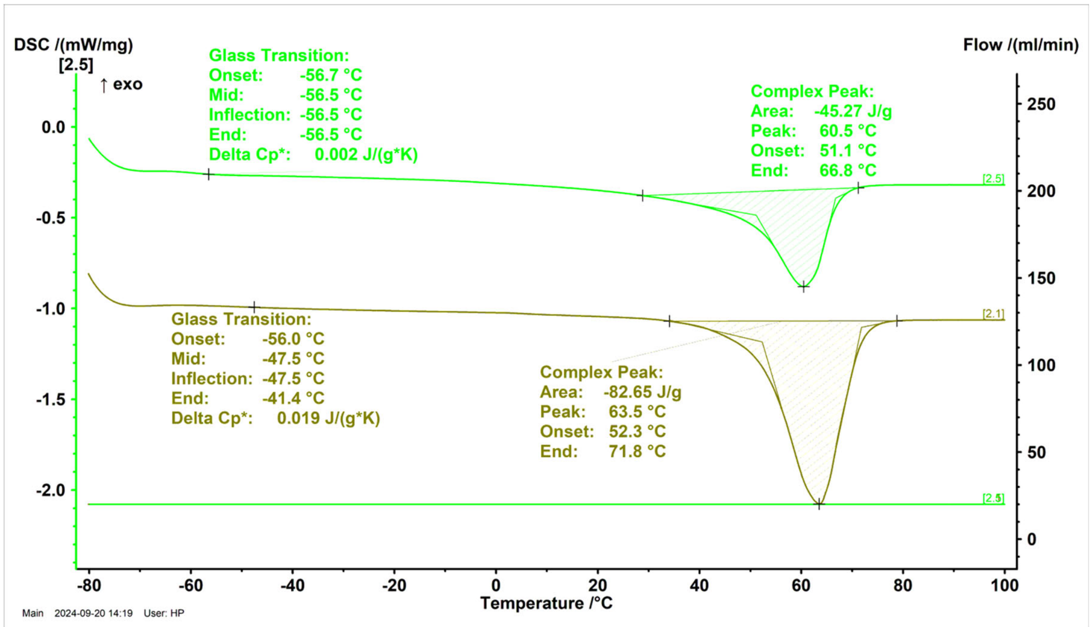

Figure S26. Differential scanning calorimetry (DSC) for PCL obtained by LiHMDS (Table 2, entry 3).

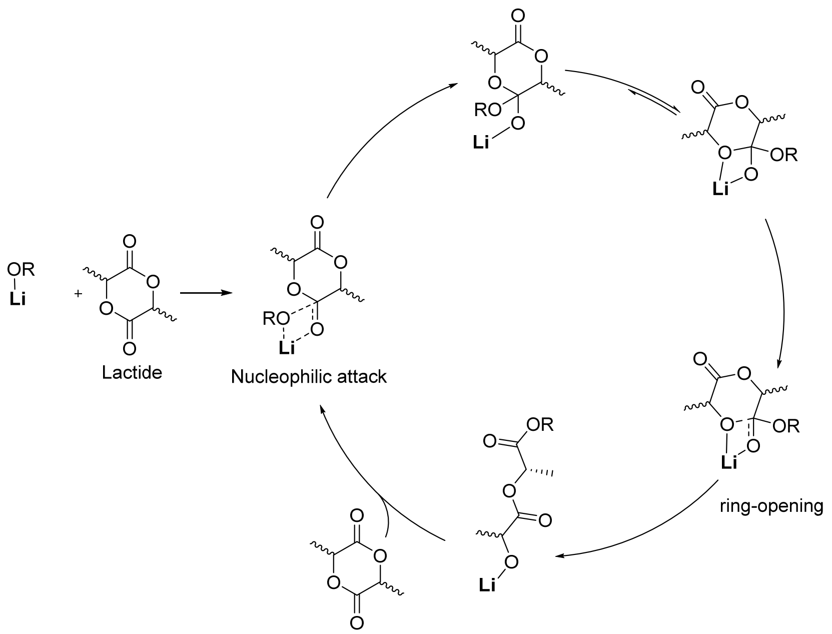

Figure S27. Proposed reaction mechanism for PLLA by LiHMDS
